# Eye-movement reveals word order effects on comparative sentences in older adults using a verb-final language

**DOI:** 10.3389/fpsyg.2024.1335536

**Published:** 2024-03-21

**Authors:** Jihyun Hwang, Seunghun J. Lee, Jee Eun Sung

**Affiliations:** ^1^Department of Communication Disorders, Ewha Womans University, Seoul, Republic of Korea; ^2^Department of Psychology and Linguistics, International Christian University, Tokyo, Japan; ^3^Department of Humanities and Social Sciences, Indian Institute of Technology Guwahati, Guwahati, India

**Keywords:** comparative sentence, sentence comprehension, verb-final language, word order, eyetracking, visual world paradigm, aging

## Abstract

**Objectives:**

This study aimed to examine age-related differences in the comprehension of Korean comparative sentences with varying word orders by employing both offline and online measures, and to investigate how variations in word order affect sentence processing across different age groups.

**Methods:**

A total of 52 monolingual native Korean speakers, 26 young adults, and 26 older adults, completed a sentence-picture-matching task under two word order conditions: comparative-first and nominative-first. Offline measures included accuracy and response time, while an online method involved eye-tracking within the Visual World Paradigm. Data analyses were performed using linear and generalized linear mixed-effects models.

**Results:**

Older adults demonstrated lower accuracy and longer response times compared to younger individuals. Distinctive fixation patterns were observed, particularly in the sentential-final phrase, across different age groups. Specifically, nominative-first sentences elicited greater target advantage scores among younger adults, whereas older adults showed higher scores in comparative-first sentences.

**Conclusion:**

The study highlights the potential of comparative sentences in elucidating age-related changes in sentence comprehension. These differences were evident not only in offline tasks but also in real-time processing, as evidenced by eye-tracking data. The findings suggest distinct processing strategies employed by young and older adults and underscore the importance of considering both syntactic and semantic cues in sentence comprehension.

## 1 Introduction

Aging leads to declined cognitive abilities and increases challenges to sentence comprehension. Studies have consistently shown age-related differences in sentence comprehension abilities between older adults and their younger counterparts in group-level experiments (Carpenter et al., 1994; Waters and Caplan, 2005; Thornton and Light, 2006). Conversely, it has also been noted that increased world knowledge and experience enhance sentence processing, particularly through the use of predictive strategies drawn from contextual cues (Kathleen Pichora-Fuller, 2008; Lash et al., 2013; Milburn et al., 2021). While the extent of impact of cognitive decline on overall sentence comprehension abilities due to aging remains debated, studies have demonstrated significant performance differences between age groups, particularly in tasks that require higher cognitive resources. Notably, older adults often exhibit decreased comprehension abilities for complex sentences compared to simple ones, such as object relative clauses (DeDe et al., 2004; Caplan et al., 2011; Amichetti et al., 2016), passive sentences (Mack et al., 2013; Sung et al., 2017), and garden path sentences with syntactic ambiguity (Waters and Caplan, 1996; Yoo and Dickey, 2017).

In our study, we explored age-related changes in sentence comprehension using comparative sentences, a common syntactic structure in the language assessments such as the Western Aphasia Battery-Revised (WAB-R; Kertesz, 2007) and the Test for Reception of Grammar (TROG-2; Bishop, 2003). Previous research has shown that children often struggle with comprehending comparative sentences, interpreting them in a simplified manner similar to simple sentences, despite the presence of explicit morphological cues for comparatives (Bishop and Bourne, 1985; Oh and Kim, 2008). While extensive research has primarily focused on children’s language development (Arii, 2013; Hackl et al., 2021), limited knowledge exists regarding the performance of people who are expected to experience difficulties in their language abilities with comparative sentences, which are inherently more complex than simple sentences.

Previous research suggests potential difficulties of older adults in comprehending comparative sentences. Obler et al. (1991) examined the effects of aging by varying the levels of syntactic complexity among participants across various age ranges. They found that older adults exhibited poorer performances compared to young adults in comprehension tasks, especially for relatively complex sentences such as comparatives. Additionally, studies have reported challenges in other groups, such as young adults and people with aphasia (PWA). For instance, Chernova et al. (2023) found that young adults experienced higher cognitive effort and reduced comprehension performance when processing comparative sentences with three object comparisons. Aithal et al. (2009) observed more significant impairments in production than in comprehension of comparative sentences among Malayalam speakers with Broca’s aphasia, while Kumar and Goswami (2013) noted differences between Hindi-speaking PWA and neurotypical adults in their comprehension of conjunctions and comparative sentences.

A simple comparative structure typically consists of two noun phrases (NPs) and an adjective as a predicate phrase (AdjP). The structure involves comparing specific qualities between two objects, determining which possesses more or less of a particular quality on a scale (Grant, 2013). For example, in the comparative sentence ‘The dog is bigger than the cat’, the NP ‘cat’ serves as the standard of comparison (the standard NP), while the NP ‘dog’ serves as the comparee NP, being compared against the standard NP (Stassen, 2013).

Korean is a predicate-final language with a typical word order of subject-object-verb (SOV). One of the linguistic characteristics of Korean is its relatively flexible word order, due to its case markers which carry linguistic information for syntactic parsing (Comrie, 1989; Sung et al., 2019). In comparative sentences, these case markers allow scrambling as long as the two NPs precede an AdjP, while preserving the meaning (Yeom, 2016). The present study employed two different word orders by varying the arrangement of the two noun phrases: nominative-first (NOM-first) and comparative-first (COMP-first). In NOM-first sentences, an NP with a nominative case marker appears at the beginning, for example, ‘The dog-_Nom_ the cat-_Comp_ big-_AdjP_’. Here, the comparee NP, ‘the dog’ is marked with the nominative case marker, ‘*ka*’, and the standard NP ‘the cat’ is attached with the comparative case marker, ‘*poda*’. Conversely, the COMP-first sentences feature an NP with a comparative case marker at the beginning, as illustrated by the sentence ‘The cat-_Comp_ the dog-_Nom_ big-_AdjP_’.

While NOM-first and COMP-first sentences maintain identical meanings, they exhibit distinct syntactic and semantic traits. Syntactically, NOM-first is considered canonical as the sentence begins with the subject marked with a nominative case marker, whereas COMP-first is regarded as non-canonical. Semantically, on the other hand, this difference can be elucidated through the isomorphic mapping hypothesis (O’Grady and Lee, 2001, 2005). It posits that sentence processing is facilitated when the structure of sentences aligns with the corresponding event sequence. In comparative sentence comprehension, this entails setting the degree of the adjective of the standard NP and then comparing it with the degree of the adjective of the comparee NP (Ha, 1999). Consequently, COMP-first sentences are considered isomorphic, where the standard NP precedes the comparee NP, aligning the syntactic structure with the event order. Conversely, NOM-first sentences are non-isomorphic, as the order of NPs does not align with the corresponding event sequence, posing more sentence processing difficulties.

It is well known that older adults demonstrate more difficulties in processing syntactically non-canonical sentences compared to canonical sentences (Sung, 2015; Sung et al., 2017). Furthermore, research has reported the benefits of isomorphic mapping sentences over non-isomorphic mapping in sentence comprehension among PWA (O’Grady and Lee, 2005), second language speakers (Chrabaszcz et al., 2022; Shin and Park, 2023). In our study, we explored a unique case where a mismatch exists between NOM-first (syntactically canonical but non-isomorphic) and COMP-first (non-canonical but isomorphic) sentences. From this mismatch in unique Korean comparative sentences, we aim to determine whether there are age-related differences in sentence processing with different word orders and to discover the interplay between semantic and syntactic cues in sentence comprehension.

To assess sentence comprehension ability, we employed both offline and online methods. Traditional offline measures include accuracy and response time, while online methods provide insights into real-time processing (DeDe and Flax, 2016). Various online techniques, such as the visual world eye-tracking paradigm (Harel-Arbeli et al., 2021; Oh et al., 2022), self-paced reading (Traxler et al., 2013; Cutter et al., 2022), and auditory moving window paradigms (Waters and Caplan, 2001), have been employed to investigate age-related differences in sentence comprehension. However, previous research has highlighted the distinction between implicit and explicit tasks in understanding these age-related changes. Real-time processing tasks often require less cognitive demand and may not reveal significant impairments, whereas offline tasks involve greater cognitive processing demands and may highlight age-related deficits. These inconsistencies between offline and online findings emphasize the necessity of using both approaches. For instance, studies on passive sentence comprehension consistently demonstrate difficulties in offline tasks across languages (Ferreira, 2003; Sung et al., 2017; Paolazzi et al., 2019; Sung et al., 2020), whereas online measures often report no significant difficulties or even better performance. Traxler et al. (2013) found that passive sentences were read faster than active ones in self-paced reading tasks, while Paolazzi et al. (2021) observed greater latency in eye-tracking studies.

Similarly, such inconsistencies arise in studies on relative clauses, with object relatives are generally considered more challenging than subject relative clauses (Waters and Caplan, 2001; Waters and Caplan, 2005; Kemper and Liu, 2007; Caplan et al., 2011). Online methods often reveal longer processing times for object relatives, particularly in cognitively demanding segments. For instance, Stine-Morrow et al. (2000) found longer reading times for object relatives in young adults, indicating decreased resource allocation for thematic role assignment. Conversely, Caplan et al. (2011) reported older adults demonstrated longer reading times on verbs, which were the most demanding parts. Waters and Caplan (2005) found longer listening times for verbs in object relative clauses but no significant age-related differences. Given these discrepancies, employing both offline and online approaches is essential for a better understanding of age-related difficulties in sentence comprehension.

The current study employed eye-tracking within the Visual World Paradigm (VWP). The VWP is a useful method for investigating auditory comprehension ability by tracking participants’ eye movements. It is based on the idea that when individuals are presented with linguistic stimuli and relevant visual displays simultaneously, their fixation is drawn to the visual referents of the words they heard as the sentence unfolds (Cooper, 1974). Therefore, it reflects an individual’s cognitive processes and offers insights into the real-time interpretation of linguistic input within the context of cognitive processing. The VWP is commonly utilized to investigate sentence-level processing, including predictive processing, resolution of lexical and syntactic ambiguity, and the effects of contextual cues (e.g., Altmann and Kamide, 1999; Huettig and Altmann, 2005; Ferreira et al., 2013; Kukona et al., 2014).

The present study investigated potential age-related differences in the comprehension of comparative sentences by varying the word order of two NPs. To achieve this goal, our objectives were: (1) to examine age-related differences in comprehension of comparative sentences and (2) to investigate how variations in word order influence this comprehension. Through offline tasks, the study sought to identify sentence comprehension difficulties across different age groups, while online tasks aimed to explore the sentence processing strategies employed by each age group and determine if any differences existed.

## 2 Method

### 2.1 Participants

A total of fifty-two monolingual native Korean speakers participated in the study, with 26 young adults (Mean age = 22.27 years, SD = 3.86, range = 19–36) and 26 older adults (Mean age 63.85 years, SD = 1.41, range = 60–78). The mean years of education were 13.69 (SD = 1.41, range 12–18) for young and 14.92 (SD = 3.16, range 9–20) for the older group, respectively. A formal power analysis for an effect size of 0.25 with an alpha of 0.05 and a power of 0.80 indicated that a total sample size of 48 was needed. The two groups were not significantly different in the level of education (*t* = −1.81, *p* = 0.078). The age ranges for each group were established based on previous research (e.g., Burda et al., 2017; Jo et al., 2023; Sung et al., 2024).

All participants self-reported no history of neurological impairments or issues with vision or hearing. Also, they scored within the normal range (age-and education-adjusted scores ≥16 percentile) on the Korean version of the Mini-Mental State Examination (Kang, 2006). Additionally, the older adults completed the Short Geriatric Depression Scale of Korean version (S-GDS; Cho et al., 1999) and the Seoul Verbal Learning Test from the Seoul Neuropsychological Screening Battery (SNSB; Kang and Na, 2003), all scoring within the normal range (S-GDS; <8 out of 15, SNSB; age-and education-adjusted scores ≥16 percentile).

### 2.2 Materials

**Figure 1:**
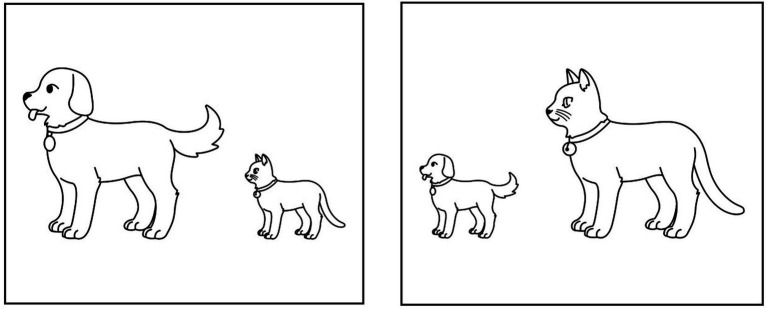
An example of the sentence-picture matching task.

In the sentence–picture matching task, the experimental stimuli comprised 64 sentences, including 32 target and 32 filler sentences. The target sentences included two types of comparative sentences with varying word orders: NOM-first and COMP-first, obtained from Park et al. (2023). Each comparative sentence consisted of two NPs and an AdjP, structured in the sequence of NP1, NP2, and AdjP. In NOM-first sentences, the comparee NP with the nominative case marker was placed at the beginning of the sentence, followed by the standard NP with the comparative case marker. Conversely, in COMP-first sentences, this order is reversed, with the standard NP preceding the comparee NP. In addition to the target sentences, filler sentences with different sentence structures were employed, including active, passive, and instrumental sentences to prevent participants from recognizing the purpose of the experiment. Table 1 presents sentence examples for each condition. A sample visual display is presented in Figure 1.

**Table 1:** Examples of sentence for each condition.

Nominative-first	Comparative-first
The dog is bigger than the cat. The dog-_Nom_ the cat-_Comp_ big-_AdjP_	Than the cat, the dog is bigger. The cat-_Comp_ the dog-_Nom_ big-_AdjP_
The cat is bigger than the dog. The cat-_Nom_ the dog-_Comp_ big-_AdjP_	Than the dog, the cat is bigger. The dog-_Comp_ the cat-_Nom_ big-_AdjP_
The pencil is longer than the pen. The pencil-_Nom_ the pen-_Comp_ long-_AdjP_	Than the pen, the pencil is longer. The pen-_Comp_ the pencil-_Nom_ long-_AdjP_
The pen is longer than the pencil. The pen-_Nom_ the pencil-_Comp_ long-_AdjP_	Than the pencil, the pen is longer. The pencil-_Comp_ the pen-_Nom_ long-_AdjP_

Nom, nominative-case marker; Comp, comparative-case marker; AdjP, adjective as a predicate.

The audio stimuli were recorded by a native Korean voice actor instructed to maintain a constant tone and speech rate. Subsequently, they were processed using Adobe Audition software, adjusting the speech rate to an average of 3.23 syllables per second, which falls within the normal range of speech rates (Kwon et al., 1998). Each stimulus was paired with two images: one representing the target sentence (target image) and another depicting the syntactic foil (foil image). For instance, one target image depicted the sentence ‘A dog is bigger than a cat,’ while the other depicted a foil showing ‘A cat is bigger than a dog.’ To prevent participants from recognizing the purpose of the experiment, filler sentences with different sentence structures, such as active and passive sentences were included. The entire experiment consisted of two sections, each containing 16 target sentences and 16 filler sentences. The locations of the target images on the monitor were counterbalanced, and the order of presenting two sections was also counterbalanced across participants, with sentences within a section were presented in a random order.

### 2.3 Experimental procedures

The experiment was conducted in a soundproof room at Ewha Womans university. Eye movements were recorded using the SMI RED (SensoMotoric Instruments), sampling at 250 Hz. Experimental development and processing were managed using the SMI Experiment Center™ 3.0 software. Eye movement tracking and analyses were conducted using SMI iView X™ system and SMI BeGaze™ software, respectively. Participants were seated approximately 60–70 cm from the monitor (1,920 pixels × 1,080 pixels), with their head and chin held in place by a headrest and chinrest to minimize head movements during the experiment. Auditory sentence stimuli were presented through a speaker next to the computer screen. Before starting the experiment, participants received an explanation of the entire process. They confirmed that the sound level was appropriate for clear hearing and were given sufficient time to become accustomed to the procedure through four practice trials.

**Figure 2:**
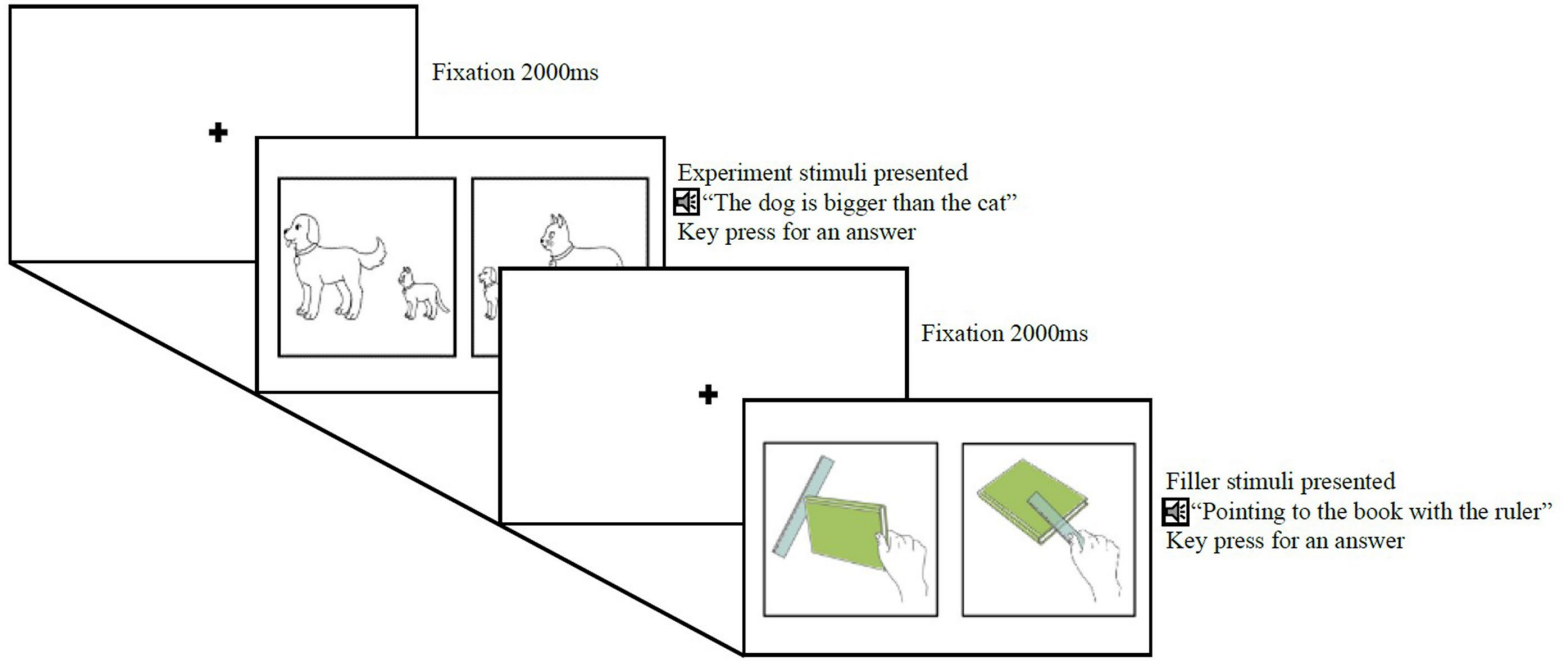
An example of the experiment procedure.

The procedure began in the following manner. A fixation cross displayed for 2,000 ms, followed by the presentation of two images on the monitor. The experimental audio file was played 500 ms after the images appeared. Following the audio presentation, participants chose one image that matched the auditory sentence by pressing the corresponding key on the keyboard. The image displays remained on the screen until participants made their selection. The next trial began when participants pressed the space bar key after choosing a picture. A short break was permitted after completing the first block, and calibration was performed before each block. The entire experiment session lasted approximately 15 to 20 minutes. Figure 2 provides an illustrative example of the experimental protocol.

### 2.4 Outcome measures and statistical analyses

Analyses were performed on both accuracy and response time for offline performances, with response time measured in milliseconds. For response time analysis, data from incorrect trials beyond ±3 standard deviations from the mean response time, as well as immediate responses (e.g., key presses before the audio file ended) were considered outliers and excluded from the analysis. Overall, data from 91.9% of young and 85.1% of older adults for NOM-first, and 89.6% of young and 82.0% of older adults for COMP-first were included.

For the eye movement analyses, three segments of phrases within each sentence (NP1, NP2, and AdjP) were examined. Data analysis was conducted within a time window that began 200 ms after the onset and ended 200 ms after the offset of each phrase (Altmann and Kamide, 2004). A fixation was counted when an eye gaze lasting more than 100 ms at the same point within 1° of visual angle, based on Dickey and Thompson (2009). Regarding eye-tracking data, incorrect trials and data not recorded were excluded. In total, data from 86.30% of young and 74.76% of older adults for NOM-first, and 86.46% of young and 73.5% of older adults for COMP-first were included.

The area of interest was defined within the area of each image (target & foil), and fixation proportions were separately measured for the target and the foil. This was derived by dividing the summed duration of fixation of one image by the total duration of fixation on both the target and the foil images. Additionally, target advantage (TA) scores were calculated from the fixation proportions, reflecting the state of fixating on the target image relative to the foil image during the critical time window. TA scores were computed by subtracting the fixation proportion of the foil image from that of the target image. Positive values of TA scores indicate more fixations on the target image than on the foil, while negative values indicate the opposite (Meyer et al., 2012). A larger positive TA score implies a higher fixation proportion for the target.

Statistical analyses were conducted using linear and generalized linear mixed-effects models, employing the lmer and glmer functions from the lme4 package (Bates et al., 2015) and lmerTest (Kuznetsova et al., 2014) in R statistical software (R Core Team, 2019). Specifically, for eye movement analyses, generalized linear mixed-effects models were fitted with a binomial distribution at NP1, NP2, and AdjP regions. In all models, group (young adults vs. older adults) and word order (COMP-first vs. NOM-first) were included as fixed effects, while participant and item were included for random effects. The reference levels were set as follows: Group = Older adults, Word order = NOM-first.

## 3 Results

### 3.1 Behavioral results

A generalized linear mixed-effects model was conducted for accuracy. The final model included fixed effects of group, word order and their interaction term, with participant and item as random intercepts. The analysis revealed that the young group performed the task significantly better than the older group (*β* = 1.8212, *SE* = 0.4699, *z* = 3.876, *p* = 0.0001). However, there was no significant main effect for word order (*β* = −0.3022, *SE* = 0.3067, *z* = −0.985, *p* = 0.3244), nor for the two-way interaction between group and word order (*β* = −0.7651, *SE* = 0.5976, *z* = −1.508, *p* = 0.132). The summary of the model of accuracy is reported in Appendix A.

For response time, a linear mixed-effects model was computed, with fixed effects of group, word order, and their interaction term, with participant and item as random intercepts. The final model revealed a significant main effect of group (*β* = −501.56, *SE* = 137.27, *t* = 3.876, *p* < 0.001), indicating that the young group responded significantly faster than the older group. However, there was no significant main effect for word order (*β* = −54.78, *SE* = 91.46, *t* = −0.599, *p* = 0.552), and the two-way interaction between group and word order was not statistically significant (*β* = 145.71, *SE* = 75.95, *t* = 1.918, *p* = 0.055). The summary of the model of response time is attached in Appendix B.

### 3.2 Eye-tracking results

A generalized linear mixed-effects model was conducted to analyze the TA score for each phrase target advantage at the NP1, NP2, and AdjP. The final model of each phrase included fixed effects of group, word order, and their interaction term, with participant and item as random intercepts.

For NP1, there were no significant main effects for group (*β* = −0.0015, *SE* = 0.1709, *z* = −0.009, *p* = 0.993), word order (*β* = −0.2591, *SE* = 0.3745, *z* = −0.692, *p* = 0.489), and the two-way interaction between group and word order (*β* = 0.3734, *SE* = 0.2427, *z* = 1.539, *p* = 0.124).

Similarly, for NP2, there were no significant main effects for group (*β* = 0.0354, *SE* = 0.1551, *z* = 0.229, *p* = 0.819), for word order (*β* = 0.0966, *SE* = 0.1652, *z* = −0.585, *p* = 0.559), and two-way interaction between group and word order (*β* = 0.0522, *SE* = 0.2198, *z* = 0.238, *p* = 0.812).

However, for AdjP, while there was no significant main effect for group (*β* = 0.4044, *SE* = 0.2176, *z* = 1.858, *p* = 0.063), there was a significant main effect for word order (*β* = 0.4671, *SE* = 0.1926, *z* = 2.424, *p* = 0.015). The TA scores for COMP-first sentences were greater than that of NOM-first sentences. Additionally, there was a significant two-way interaction between group and word order (*β* = −0.7469, *SE* = 0.2440, *z* = −3.061, *p* = 0.002), indicating that the effect of word order on the TA score varied significantly between the young and older groups. Specifically, while the TA scores for NOM-first sentences were greater than that of COMP-first sentences in the young group, the pattern was reversed in the older group, where the TA scores for COMP-first sentences were greater than that of NOM-first sentences. The details of the models of target advantage are reported in Appendix C.

## 4 Discussion

This study investigated age-related differences in sentence comprehension using comparative sentences by varying the word order of the two NPs. Remarkably, comparative sentences proved sensitive enough to detect aging effects in a predicate-final language. Older adults exhibited considerably lower accuracy and longer response times, aligning with previous research indicating age-related declines in cognitive resources and processing efficiency (Stine-Morrow et al., 2000; Waters and Caplan, 2005; Caplan et al., 2011).

In the online eye-tracking analyses, no significant differences were found in TA scores for NP1 and NP2 between age groups. These findings may be explained by participants instinctively directed their gaze toward relevant nouns upon hearing the experimental auditory stimuli, alternating between target and foil images until reaching the AdjP (Cooper, 1974). Consequently, no clear preference was evident between the target and foil images in these initial segments. However, distinctive patterns emerged in the processing of the AdjP. NOM-first sentences resulted in greater TA scores among young adults, while older adults tended to exhibit greater scores with COMP-first sentences. In predicate-final languages, the predicate plays a crucial role in integrating all linguistic information; therefore, significant outcomes were revealed in the segment. This pattern highlights the differential impact of word order in comparative sentence processing across age groups.

Referring to the isomorphic mapping theory (O’Grady and Lee, 2001, 2005), NOM-first and COMP-first sentences possess distinct syntactic and semantic traits. NOM-first sentences are syntactically canonical but non-isomorphic, while COMP-first sentences demonstrate the opposite pattern. This distinction suggests different use of strategies in comparative sentence processing between the two age groups. The higher scores observed for NOM-first sentences among young adults indicate a reliance on syntactic canonicity over semantic cues in their comprehension of comparative sentences. In contrast, older adults demonstrated a preference for semantic cues, suggesting that isomorphic sentences, which the structures align with the event order, might induce their better performance.

Interestingly, similar patterns were observed in the findings of O’Grady and Lee (2005). A group of PWA conducted English instrumental sentence comprehension tasks with manipulated word orders. They showed significant difficulties in comprehending non-isomorphic but syntactically canonical sentences. Notably, our study observed a similar pattern among older adults, suggesting potential parallels with individuals facing language difficulties.

Expanding on these findings, Oh and Kim (2008) examined the comprehension abilities of children aged four to six using comparative sentences in different word orders. The study observed significant better performance in COMP-first sentences, indicating that the children found it easier to comprehend isomorphic sentences. Similarly, Seol and Jeon (2022) reported preferences for COMP-first in both comprehension and production tasks over NOM-first sentences among children. Although these prior studies primarily targeted children who were in the middle of language acquisition, it is intriguing to observe a comparable outcome even with older adults.

In conclusion, our research highlights the potential of even comparative sentences to reveal age-related changes in sentence comprehension. These age-related declines occurred not only in offline tasks but also in real-time processing, as evidenced by the visual world eye-tracking paradigm. Moreover, the study uncovered distinct fixation patterns between the young and older individuals through word order manipulations, indicating different usage of processing strategies across age groups. The significance of our study is emphasized by the observed shift in reliance from syntactic to semantic cues with age, along with the importance of considering the interplay between these linguistic elements even in simple sentence comprehension. Further research is needed to explore the underlying mechanisms of comparative sentences comprehension related to age-related changes using a range of online methodologies.

## Data availability statement

The raw data supporting the conclusions of this article will be made available by the authors, without undue reservation.

## Ethics statement

The studies involving humans were approved by the Institutional Review Board on Human Subjects of Ewha Womans University (ewha-202208-0024-02, August 2022). The studies were conducted in accordance with the local legislation and institutional requirements. The participants provided their written informed consent to participate in this study. Written informed consent was obtained from the individual(s) for the publication of any potentially identifiable images or data included in this article.

## Author contributions

JH: Writing – review & editing, Writing – original draft, Visualization, Validation, Software, Project administration, Methodology, Investigation, Formal analysis, Data curation, Conceptualization. SL: Writing – review & editing, Resources. JS: Writing – review & editing, Validation, Supervision, Resources, Project administration, Funding acquisition, Formal analysis, Conceptualization.
