# Supplementary material for: Eye-movement reveals word order effects on comparative sentences in older adults using a verb-final language
Source: Front Psychol. 2024 Mar 21;15:1335536. doi: 10.3389/fpsyg.2024.1335536 (PMC11002905; doi:10.3389/fpsyg.2024.1335536)
Supplement: Supplementary file 1 [file Data_Sheet_1.docx]

**Appendix A**

Summary of the Generalized Linear Mixed-Effects Model of Accuracy

| **Predictors** | ***β*** | ***SE*** | ***z*** | ***p*** |
| --- | --- | --- | --- | --- |
| Intercept | 2.5852 | 0.2752 | 9.392 | .000*** |
| Group | 1.8212 | 0.4699 | 3.876 | .000*** |
| Word order | -0.3022 | 0.3067 | -0.985 | .324 |
| Group x Word order | -0.7651 | 0.5976 | -1.508 | .132 |
| *Note.* R model equation: Accuracy ~ Group * Wordorder + (1 \| Participant) + (1 \| Item).  Reference levels are as follows: Group = older group, Word order = NOM-first. SE = standard error.  ****p* < .001. | | | | |

**Appendix B**

Summary of the Linear Mixed-Effects Model of Response Time

| **Predictors** | ***β*** | ***SE*** | ***t(df)*** | ***p*** |
| --- | --- | --- | --- | --- |
| Intercept | 4708.84 | 110.02 | 42.801(76.97) | .000*** |
| Group | -501.56 | 137.27 | -3.654(58.88) | .000*** |
| Word order | -54.78 | 91.46 | -0.599(58.88) | .552 |
| Group x Word order | 145.71 | 75.95 | 1.918(1339.79) | .055 |
| *Note.* R model equation: Response time ~ Group * Wordorder + (1 \| Participant) + (1 \| Item). Reference levels are as follows: Group = older group, Word order = NOM-first. SE = standard error.  ****p* < .001. | | | | |

**Appendix C**

Summary of the Generalized Linear Mixed Effects Models of Target Advantage

| **Predictors** | ***β*** | ***SE*** | ***z*** | ***p*** |
| --- | --- | --- | --- | --- |
| NP1 | | | | |
| Intercept | -0.0794 | 0.2643 | -0.300 | .764 |
| Group | -0.0015 | 0.1709 | -0.009 | .993 |
| Word order | -0.2591 | 0.3745 | -0.692 | .489 |
| Group x Word order | 0.3734 | 0.2427 | 1.539 | .124 |
| NP2 | | | | |
| Intercept | 0.0370 | 0.1164 | 0.318 | .751 |
| Group | 0.0354 | 0.1551 | 0.229 | .819 |
| Word order | -0.0966 | 0.1652 | -0.585 | .559 |
| Group x Word order | 0.0522 | 0.2198 | 0.238 | .812 |
| AdjP | | | | |
| Intercept | 0.5942 | 0.1635 | 3.634 | .000*** |
| Group | 0.4044 | 0.2176 | 1.858 | .063 |
| Word order | 0.4671 | 0.1926 | 2.424 | .015* |
| Group x Word order | -0.7469 | 0.2440 | -3.061 | .002** |
| *Note.* R model equations: NP1 Target advantage ~ Group * Wordorder + (1 \| Participant) + (1 \| Item); NP2 Target advantage ~ Group * Wordorder + (1 \| Participant) + (1 \| Item); AdjP Target advantage ~ Group * Word order + (1 \| Participant) + (1 \| Item).  Reference levels are as follows: Group = older group, Word order = Nom-first. NP = noun phrase; AdjP = adjective as predicate, SE = standard error.  * *p* < .05, ***p* < .01, ****p* < .001. | | | | |
